# Ticagrelor monotherapy in patients with concomitant diabetes mellitus and chronic kidney disease: a post hoc analysis of the GLOBAL LEADERS trial

**DOI:** 10.1186/s12933-020-01153-x

**Published:** 2020-10-16

**Authors:** Chao Gao, Mariusz Tomaniak, Kuniaki Takahashi, Hideyuki Kawashima, Rutao Wang, Hironori Hara, Masafumi Ono, Gilles Montalescot, Scot Garg, Michael Haude, Ton Slagboom, Pascal Vranckx, Marco Valgimigli, Stephan Windecker, Robert-Jan van Geuns, Christian Hamm, Philippe Gabriel Steg, Yoshinobu Onuma, Dominick J. Angiolillo, Patrick W. Serruys

**Affiliations:** 1 Department of Cardiology, Xijing hospital, Xi’an, China; 2 Department of Cardiology, Radboud University, Nijmegen, The Netherlands; 3 First Department of Cardiology, Medical University of Warsaw, Warsaw, Poland; 4 Erasmus Medical Center, Erasmus University, Rotterdam, The Netherlands; 5 Amsterdam UMC, University of Amsterdam, Amsterdam, The Netherlands; 6 Sorbonne University, ACTION Study Group, Institute of Cardiology, Pitié-Salpêtrière Hospital, Paris, France; 7 Department of Cardiology, Royal Blackburn Hospital, Blackburn, UK; 8 Department of Cardiology, Rheinland Klinikum Neuss, Lukaskrankenhaus, Neuss, Germany; 9 OLVG, Amsterdam, Netherlands; 10 Department of Cardiology and Critical Care Medicine, Hartcentrum Hasselt, Jessa Ziekenhuis, Hasselt, Belgium; 11 Department of Cardiology, Bern University Hospital, Bern, Switzerland; 12 Kerckhoff Heart Center, Bad Nauheim, Germany; 13 FACT, French Alliance for Cardiovascular Trials, Paris, France; 14 Hôpital Bichat, AP-HP, Paris, France; 15 Department of Cardiology, National University of Ireland Galway, Galway, Ireland; 16 Division of Cardiology, University of Florida College of Medicine, Jacksonville, FL USA; 17 NHLI, Imperial College London, London, UK; 18 Interventional Medicine and Innovation, National University of Ireland Galway, P.O. University Road, Galway, H91 TK33 Ireland

**Keywords:** Chronic kidney disease, Diabetes mellitus, Percutaneous coronary intervention, DAPT, Ticagrelor, Aspirin-free antiplatelet strategies

## Abstract

**Background:**

Patients with both diabetes mellitus (DM) and chronic kidney disease (CKD) are a subpopulation characterized by ultrahigh ischemic and bleeding risk after percutaneous coronary intervention. There are limited data on the impact of ticagrelor monotherapy among these patients.

**Methods:**

In this post hoc analysis of the GLOBAL-LEADERS trial, the treatment effects of the experimental (one-month dual-antiplatelet therapy [DAPT] followed by 23-month ticagrelor monotherapy) versus the reference regimen (12-month DAPT followed by 12-month aspirin alone) were analyzed according to DM/CKD status. The primary endpoint was a composite endpoint of all-cause death or new Q-wave myocardial infarction at 2-years. The patient-oriented composite endpoint (POCE) was defined as the composite of all-cause death, any stroke, site-reported MI and any revascularization, whereas net adverse clinical events (NACE) combined POCE with BARC type 3 or 5 bleeding events.

**Results:**

At 2 years, the DM + /CKD + patients had significantly higher incidences of the primary endpoint (9.5% versus 3.1%, adjusted HR 2.16; 95% CI [1.66–2.80], p < 0.001), BARC type 3 or 5 bleeding events, stroke, site-reported myocardial infraction, all revascularization, POCE, and NACE, compared with the DM-/CKD- patients. Among the DM + /CKD + patients, after adjustment, there were no significant differences in the primary endpoints between the experimental and reference regimen; however, the experimental regimen was associated with lower rates of POCE (20.6% versus 25.9%, HR 0.74; 95% CI [0.55–0.99], p = 0.043, p_interaction_ = 0.155) and NACE (22.7% versus 28.3%, HR 0.75; 95% CI [0.56–0.99], p = 0.044, p_interaction_ = 0.310), which was mainly driven by a lower rate of all revascularization, as compared with the reference regimen. The landmark analysis showed that while the experimental and reference regimen had similar rates of all the clinical endpoints during the first year, the experimental regimen was associated with significantly lower rates of POCE (5.8% versus 11.0%, HR 0.49; 95% CI [0.29–0.82], p = 0.007, p_interaction_ = 0.040) and NACE (5.8% versus 11.2%, HR 0.48; 95% CI [0.29–0.82], p = 0.007, p_interaction_ = 0.013) in the second year.

**Conclusion:**

Among patients with both DM and CKD, ticagrelor monotherapy was not associated with lower rates of all-cause death or new Q-wave, or major bleeding complications; however, it was associated with lower rates of POCE and NACE. These findings should be interpreted as hypothesis-generating.

*Clinical Trial Registration:* ClinicalTrials.gov (NCT01813435).

## Background

Patients with coronary artery disease (CAD) and concomitant diabetes mellitus (DM) or chronic kidney disease (CKD) are more susceptible to major adverse cardiovascular and cerebrovascular events [1]. Moreover, the presence of these risk factors is also associated with an increased risk of bleeding complications [2, 3]. DM and CKD frequently co-exist and given that DM is a well-established risk factor for renal dysfunction [2, 4], it is predicted that nearly 25% of DM patients have CKD [5].

Previously, a subgroup analysis of the PLATO study has demonstrated that in the acute coronary syndrome (ACS) population, those who had both DM and CKD were associated with a drastically unfavorable prognosis compared to those having one or neither of these comorbidities [6], and among the patients with both DM and CKD, the combination of ticagrelor with aspirin substantially reduced cardiovascular death, myocardial infarction (MI), or stroke compared with clopidogrel plus aspirin; however, the dual antiplatelet therapy (DAPT) with ticagrelor had a higher rate of TIMI non-CABG-related major bleeding events.

In an attempt to mitigate bleeding risk while preserving the anti-ischemic efficacy, the “aspirin-free” antiplatelet strategy has been advocated [7–10]. The first and largest trial to date evaluating this concept -GLOBAL LEADERS, failed to show the superiority of ticagrelor monotherapy over standard DAPT in an all-comer patient population (in terms of all-cause mortality or new Q-wave MI) [7]. Nevertheless, understanding the impact of ticagrelor monotherapy after PCI in patients with DM and CKD in this large all-comer contemporary trial is still of clinical interest. The ever-growing prevalence of CKD in patients with DM [11, 12] underscores the need to specifically investigate the effects of different antiplatelet strategies in these ultrahigh risk patients.

On this background, here we report the results of a post hoc analysis of the GLOBAL LEADERS trial, in which we compared the outcomes of patients according to the presence or absence of DM and CKD, and also analyzed the effects of the experimental strategy (1-month DAPT followed by 23-month ticagrelor monotherapy) compared to the reference strategy (12-month DAPT followed by aspirin monotherapy for 12 months) after PCI in such defined subgroups.

### Methods

**Fig. 1:**
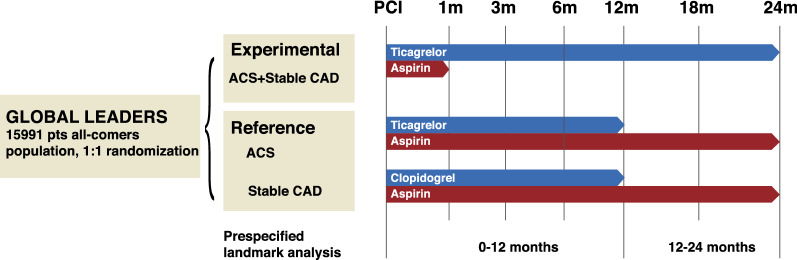
Illustration of the antiplatelet strategy in the GLOBAL LEADERS trial

The present study is a post hoc subgroup analysis of the GLOBAL LEADERS trial. GLOBAL LEADERS trial is a prospective, multi-center, randomized controlled trial (NCT01813435), which enrolled a total of 15,991 patients at 130 hospitals in 18 countries (Europe, Asia, Brazil, Australia and, Canada) between July 2013 and November 2015, and aimed to evaluate two antiplatelet strategies after PCI using bivalirudin and biolimus A9-eluting stents (Biomatrix) in an all-comers population [13]. Details of the study have been previously described. In brief, the experimental treatment strategy comprised aspirin 75–100 mg once daily in combination with ticagrelor 90 mg twice daily for one month, followed by ticagrelor 90 mg twice daily alone for 23 months (irrespective of clinical presentation). The reference treatment strategy included aspirin 75–100 mg daily in combination with either clopidogrel 75 mg once daily in patients with stable CAD or ticagrelor 90 mg twice daily in patients with ACS for 1 year, followed by aspirin 75–100 mg once daily alone for the following 12 months (from 12 to 24 months after PCI). Patients were followed up at 30 days and 3, 6, 12, 18 and 24 months after the index procedure. An illustration of the antiplatelet strategy used in the trial is shown in Fig. 1.

The trial was approved by the institutional review board at each center and followed the ethical principles of the Declaration of Helsinki. All patients provided written informed consent prior to participation in the trial.

### Patients

The GLOBAL LEADERS trial randomized 15,991 participants -23 patients withdrew consent and requested the deletion of their data from the database [7] -DM and CKD status was unavailable in 96 patients, leaving 15,872 patients (99.2%) for the present analyses. Patients with DM or CKD were pre-specified subgroups of the GLOBAL LEADERS study [13]. However, the analyses of current analyses were not pre-specified. As pre-specified in the trial protocol, CKD was defined at the time of randomization, using an eGFR cut-off of 60 ml/min/1.73 m^2^ (stage III to V CKD by KDIGO classification), calculated according to the Modification of Diet in Renal Disease (MDRD) equation [14]. A sensitivity analysis was performed by defining CKD using an eGFR cut-off of 90 ml/min/1.73 m^2^ (equivalent to the stage II to V CKD by KDIGO classification, results shown in Additional file 1: Table S3). The status of DM was site-reported and defined at the time of randomization [13]. The PRECISE-DAPT score was calculated by the online calculator [15].

### Outcomes

The events definitions have been reported previously [16]. The primary endpoint was a composite of all-cause mortality or new Q-wave myocardial infarction (MI). The key secondary safety endpoint was investigator-reported Bleeding Academic Research Consortium (BARC) type 3 or 5 bleeding [17]. Other secondary endpoints included: individual components of the primary endpoint (all-cause death, new Q-wave MI), individual components of key secondary safety endpoint (BARC defined bleeding type 3 or type 5 bleeding), any stroke, site-reported MI, any revascularization, target vessel revascularization (TVR), definite stent thrombosis (ST) defined according to the Academic Research Consortium criteria [18]. The site-reported MI was defined according to the Third Universal Myocardial Infarction definition, as pre-specific in the study protocol [13]. The patient-oriented composite endpoint (POCE)—advocated by Academic Research Consortium (ARC)-2, and net adverse clinical events (NACE) were explored up to two years [17, 19]. POCE was defined as the composite of all-cause death, any stroke, site-reported MI (including periprocedural or spontaneous with ST elevation MI [STEMI] or non-ST-segment elevation MI [NSTEMI]) and any revascularization (re-PCI or coronary artery bypass graft surgery [CABG] in the target or non-target vessel) [19], whereas NACE combined POCE with BARC type 3 or 5 bleeding events. Composite endpoints were analyzed hierarchically and the individual components of the composite endpoints were reported non-hierarchically.

### Statistical analysis

All the analyses were performed by the intention-to-treat principle. Continuous variables with normal distribution are expressed as mean ± standard deviation and those with skewed distribution are expressed as median ± interquartile range. Categorical variables are presented as counts and percentages. Means of 2 continuous variables were compared by independent samples Student’s t-test or Mann–Whitney U test when appropriate. The frequencies of categorical variables were compared using Fisher’s exact test. Survival was estimated by the Kaplan–Meier method. The impacts of CKD and DM on outcomes were assessed in the multivariable Cox proportional hazards model. The covariates in the multivariable model included age, sex, body mass index (BMI), clinical presentation (ACS versus stable CAD), stroke, peripheral vascular disease (PVD), chronic obstructive pulmonary disease (COPD), hypertension, previous PCI, hypercholesterolemia, current smoking status, treatment regimen (experimental versus. reference regimen), complex PCI, ACEI or ARB, beta-blockade, statin, Paris thrombotic risk score, and Paris bleeding risk score. A sensitivity analysis was conducted by adjusting the Cox proportional hazards model with the Propensity score (Propensity score was calculated by including all variables in Table 1). The detailed methods to calculate Propensity score and the results of the sensitivity analysis were shown in Additional file 1: Methods and Table S8. Cox proportionality assumptions were checked by using the Schoenfeld residuals against the transformed time and the assumptions were met in all models. Landmark analyses were performed at 365 days of follow-up, which was based on the prespecified landmark point in the GLOBAL LEADERS design. So far, there have been 24 subgroup analyses of the GLOBAL LEADERS trial. However, taking into account the post hoc and exploratory nature of the analysis, there was no formal correction for multiple testing [20]. Analyses were performed using R-project (R Foundation, Vienna, Austria). A two-sided p value less than 0.05 was considered as statistical significance.

**Table 1:** Baseline Characteristics according to DM/CKD status

Characteristic	DM (-) CKD (-)	DM ( +) CKD (-)	DM (-) CKD ( +)	DM ( +) CKD ( +)
	n = 10,513	n = 3189	n = 1332	n = 838
Age, years (SD)	63.0 (10.2)	65.0 (9.2)	71.5 (9.5)	71.3 (8.8)*
Male	8387/10,513 (79.8%)	2445/3189 (76.7%)	830/1332 (62.3%)	518/838 (61.8%)*
Mean body-mass index, kg/m^2^ (SD)	27.65 (4.3)	29.59 (5.0)	28.01 (4.5)	29.91 (5.0)*
Medical history				
Insulin-dependent diabetes mellitus	0/10,513 (0.0%)	869/3155 (27.3%)	0/1332 (0.0%)	352/836 (42.0%)
Hypertension	7047/10,471 (67.3%)	2721/3185 (85.4%)	1118/1329 (84.1%)	770/838 (91.9%)*
Hypercholesterolemia	6771/10,196 (64.4%)	2421/3085 (75.9%)	879/1286 (66.0%)	634/813 (75.7%)*
Current smoker	3135/10,513 (29.8%)	686/3189 (21.5%)	207/1332 (15.5%)	110/838 (13.1%)*
Previous stroke	209/10,501 (2.0%)	120/3182 (3.8%)	48/1330 (3.6%)	44/838 (5.3%)*
Previous peripheral vascular disease	480/10,433 (4.6%)	273/3158 (8.6%)	121/1317 (9.1%)	126/826 (15.0%)*
Chronic obstructive pulmonary disease	485/10,474 (4.6%)	179/3174 (5.6%)	86/1327 (6.5%)	69/828 (8.2%)*
Previous myocardial infarction	2265/10,487 (21.5%)	815/3176 (25.6%)	344/1330 (25.8%)	269/835 (32.1%)*
Previous PCI	3107/10,504 (29.6%)	1249/3186 (39.2%)	471/1331 (35.4%)	372/838 (44.4%)*
Previous CABG	477/10,506 (4.5%)	266/3185 (8.4%)	91/1331 (6.8%)	107/838 (12.8%)*
Previous bleeding	59/10,504 (0.6%)	18/3181 (0.6%)	15/1331 (1.1%)	6/838 (0.7%)
Clinical presentation				
Stable coronary artery disease	5298/10,513 (50.4%)	1913/3189 (60.0%)	690/1332 (51.8%)	514/838 (61.3%)
Acute coronary syndrome	5215/10,513 (49.6%)	1276/3189 (40.0%)	642/1332 (48.2%)	324/838 (38.7%)
Complex PCI	2976/10,513 (28.3%)	934/3189 (29.3%)	377/1332 (28.3%)	263/838 (31.4%)
Multivessel PCI	2216/10,513 (21.1%)	671/3189 (21.0%)	282/1332 (21.2%)	189/838 (22.6%)
Lesion treated ≥ 3	851/10,513 (8.1%)	266/3189 (8.3%)	113/1332 (8.5%)	68/838 (8.1%)
Stent implanted ≥ 3	1793/10,513 (17.1%)	568/3189 (17.8%)	235/1332 (17.6%)	162/838 (19.3%)
Bifurcation PCI with ≥ 2 stents	323/10,513 (3.1%)	88/3189 (2.8%)	31/1332 (2.3%)	28/838 (3.3%)
Total stent length >60 mm	1346/10,513 (12.8%)	437/3189 (13.7%)	180/1332 (13.5%)	106/838 (12.7%)
Total Stent Length (SD)	35.2 (25.1)	36.0 (25.2)	35.7 (25.8)	36.3 (26.2)
Medications on discharge				
ACE-inhibition and/or ARB	6346/10,450 (60.4%)	1986/3162 (62.3%)	730/1320 (54.8%)	457/826 (54.5%)
Beta-blockade	8194/10,452 (77.9%)	2577/3163 (80.8%)	1069/1321 (80.3%)	669/826 (79.8%)
Statin	9718/10,459 (92.4%)	2916/3168 (91.4%)	1212/1322 (91.0%)	764/827 (91.2%)
Paris bleeding risk score [31] (IQR)	3 (2,4)	3 (2,4)	6 (5,7)	6 (5,7)*
Paris thrombotic risk score (IQR)	2 (0,4)	3 (2,4)	4 (2,7)	5 (4,7)*
Paris bleeding risk score ≥ 8	100/10,039 (1.0%)	41/3060 (1.3%)	269/1288 (20.9%)	189/803 (23.5%)*
Paris thrombotic risk score ≥ 5	140/10,506 (1.3%)	655/3185 (20.8%)	243/1331 (18.3%)	615/838 (73.4%)*
PRECISE DAPT score [15] (IQR)	14 (9,19)	15 (10,20)	27 (23,32)	29 (24,34)*
PRECISE DAPT score ≥ 25	731/9849 (7.4%)	323/3007 (10.7%)	846/1266 (66.8%)	567/799 (71.0%)*
Antiplatelet therapy				
Reference treatment strategy	5297/10,513 (50.4%)	1575/3189 (49.4%)	662/1332 (49.7%)	410/838 (48.9%)
Experimental treatment strategy	5216/10,513 (49.6%)	1614/3189 (50.6%)	670/1332 (50.3%)	428/838 (51.1%)

Data are n/N (%), unless otherwise specified. Denominators vary because medical history data were incomplete

^*^The comparison between DM-/CKD + and DM + /CKD + was statistically significant

## Results

### Patients and outcomes according to DM and CKD status

A total of 15,872 patients from the GLOBAL LEADERS trial population were classified according to the DM and CKD status as follows: DM-/CKD- (n = 10,513), DM + /CKD- (n = 3189), DM-/CKD + (n = 1332), and DM + /CKD + (n = 838). Baseline characteristics are presented in Table 1. Patients with DM + /CKD + were older, more often had a prior history of revascularization (PCI or CABG), previous stroke, previous MI, PVD, COPD. In DM + /CKD + patients, the percentages of patients who had a Paris bleeding risk score ≥ 8 (23.5%), thrombotic risk score ≥ 5 (73.4%), and PRECISE-DAPT score ≥ 25 (71.0%) were higher compared with DM-/CKD- patients.

**Fig. 2:**
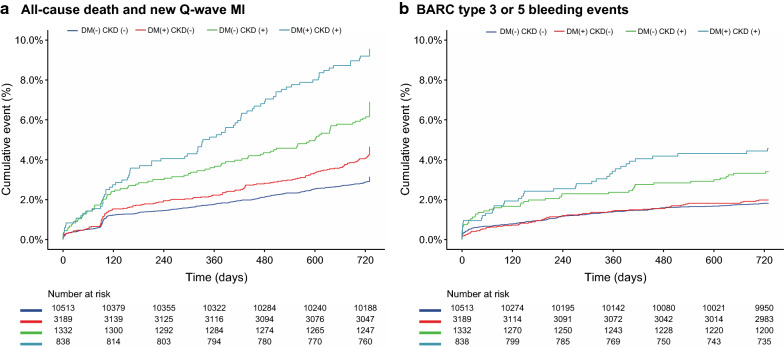
Clinical events shown by Kaplan–Meier curves. **a** All-cause mortality and new Q-wave MI; **b** Bleeding Academic Research Consortium (BARC)–defined type 3 or 5 bleeding events;

The DM + /CKD + patients had a 2.16-fold higher incidence of the primary endpoint at 24 months, compared with the DM-/CKD- individuals (9.5% versus 3.1%, adjusted HR 2.16; 95% CI [1.66–2.80], Table 2). The DM-/CKD + (6.9%, adjusted HR 1.53; 95% CI [1.20–2.80]) and DM + /CKD- patients (4.6%, adjusted HR 1.40; 95% CI [1.15–1.72]) had intermediate risk profile. With the DM + /CKD + patients exhibiting the highest risk, the hazard ratio gradually decreased in the order of DM-/CKD + , DM + /CKD- and DM-/CKD- (P_Trend_ < 0.001; Fig. 2 and Table 2). Similar trends were observed in the key secondary endpoint (Bleeding Academic Research Consortium [BARC] type 3 or 5 bleeding), and other secondary endpoints including all-cause mortality, stroke, MI, revascularization, TVR, POCE, and NACE (Table 2).

**Table 2:** Clinical outcomes according to DM/CKD subgroup

	DM (-) CKD (-)		DM ( +) CKD (-)		DM (-) CKD ( +)		DM ( +) CKD ( +)	P_trend_
	n = 10,513	HR (95% CI)	HR (95% CI)	n = 1332	HR (95% CI)	n = 838	HR (95% CI)	
All-cause mortality or New Q-wave MI	330 (3.1%)	1.00 (Ref)	1.32 (1.07–1.61)	92 (6.9%)	1.54 (1.21–2.49)	80 (9.5%)	1.91 (1.47–2.49)	< 0.001
All-cause mortality	226 (2.1%)	1.00 (Ref)	1.37 (1.08–1.75)	74 (5.6%)	1.67 (1.27–2.81)	67 (8%)	2.09 (1.55–2.81)	< 0.001
New Q-wave MI	108 (1%)	1.00 (Ref)	1.29 (0.9–1.86)	19 (1.4%)	1.19 (0.72–2.3)	13 (1.6%)	1.26 (0.69–2.3)	0.257
Stroke	78 (0.7%)	1.00 (Ref)	1.86 (1.28–2.71)	18 (1.4%)	1.17 (0.68–3.48)	19 (2.3%)	2.04 (1.19–3.48)	0.013
MI	273 (2.6%)	1.00 (Ref)	1.48 (1.18–1.85)	52 (3.9%)	1.61 (1.18–3.49)	53 (6.3%)	2.54 (1.85–3.49)	0.001
Any Revascularization	917 (8.7%)	1.00 (Ref)	1.26 (1.11–1.43)	129 (9.7%)	1.17 (0.96–1.91)	113 (13.5%)	1.55 (1.26–1.91)	< 0.001
TVR	466 (4.4%)	1.00 (Ref)	1.5 (1.27–1.78)	71 (5.3%)	1.28 (0.99–2.37)	67 (8.0%)	1.8 (1.38–2.37)	< 0.001
Definite stent thrombosis	82 (0.8%)	1.00 (Ref)	1.08 (0.69–1.7)	13 (1.0%)	1.43 (0.78–2.23)	6 (0.7%)	0.94 (0.4–2.23)	0.569
MACE	394 (3.7%)	1.00 (Ref)	1.43 (1.19–1.71)	101 (7.6%)	1.4 (1.11–2.42)	93 (11.1%)	1.9 (1.49–2.42)	< 0.001
POCE	1242 (11.8%)	1.00 (Ref)	1.3 (1.17–1.45)	219 (16.4%)	1.3 (1.12–2.06)	194 (23.2%)	1.75 (1.49–2.06)	< 0.001
NACE	1360 (12.9%)	1.00 (Ref)	1.27 (1.15–1.41)	245 (18.4%)	1.29 (1.12–2.03)	213 (25.4%)	1.74 (1.49–2.03)	< 0.001
BARC 3 or 5 bleeding	188 (1.8%)	1.00 (Ref)	1.05 (0.78–1.41)	44 (3.3%)	1.18 (0.83–2.39)	37 (4.4%)	1.64 (1.12–2.39)	0.02
BARC 5 bleeding	27 (0.3%)	1.00 (Ref)	0.51 (0.2–1.3)	8 (0.6%)	1.16 (0.5–2.09)	5 (0.6%)	0.71 (0.24–2.09)	0.704
BARC 3 bleeding	173 (1.6%)	1.00 (Ref)	1.12 (0.83–1.52)	41 (3.1%)	1.22 (0.85–2.71)	35 (4.2%)	1.84 (1.25–2.71)	0.005
BARC 3a bleeding	77 (0.7%)	1.00 (Ref)	1.31 (0.85–2.01)	25 (1.9%)	1.64 (1.02–2.63)	13 (1.6%)	1.42 (0.76–2.63)	0.054
BARC 3b bleeding	74 (0.7%)	1.00 (Ref)	1.01 (0.62–1.64)	17 (1.3%)	1.29 (0.73–3.71)	14 (1.7%)	2.02 (1.1–3.71)	0.043
BARC 3c bleeding	38 (0.4%)	1.00 (Ref)	0.78 (0.37–1.65)	3 (0.2%)	0.38 (0.11–4.56)	10 (1.2%)	2.11 (0.98–4.56)	0.524
BARC 2 bleeding	489 (4.7%)	1.00 (Ref)	1 (0.83–1.21)	82 (6.2%)	1.07 (0.84–1.52)	54 (6.4%)	1.14 (0.85–1.52)	0.388
BARC 2, 3 or 5 bleeding	647 (6.2%)	1.00 (Ref)	1.01 (0.86–1.19)	118 (8.9%)	1.11 (0.9–1.62)	84 (10%)	1.27 (1–1.62)	0.063

Data are n/N (%), unless otherwise specified

MI, Myocardial ischemia

TVR, Target vessel revascularization

MACE, all-cause death, any stroke, or non-fatal new Q-wave MI

POCE, all-cause death, any stroke, any myocardial infarction or any revascularization

BARC, Bleeding Academic Research Consortium

NACE, POCE and BARC 3 or 5 bleeding

Adjusted to age, sex, body mass index (BMI), clinical presentation (ACS versus stable CAD), stroke, peripheral vascular disease (PVD), chronic obstructive pulmonary disease (COPD), previous PCI, hypercholesterolemia, hypertension, current smoking status, treatment regimen (experimental versus. reference regimen), complex PCI, ACEI or ARB, beta-blockade, statin, Paris thrombotic risk score, and Paris bleeding risk score

### Outcomes of experimental versus reference regimen according to CKD and DM status

**Fig. 3:**
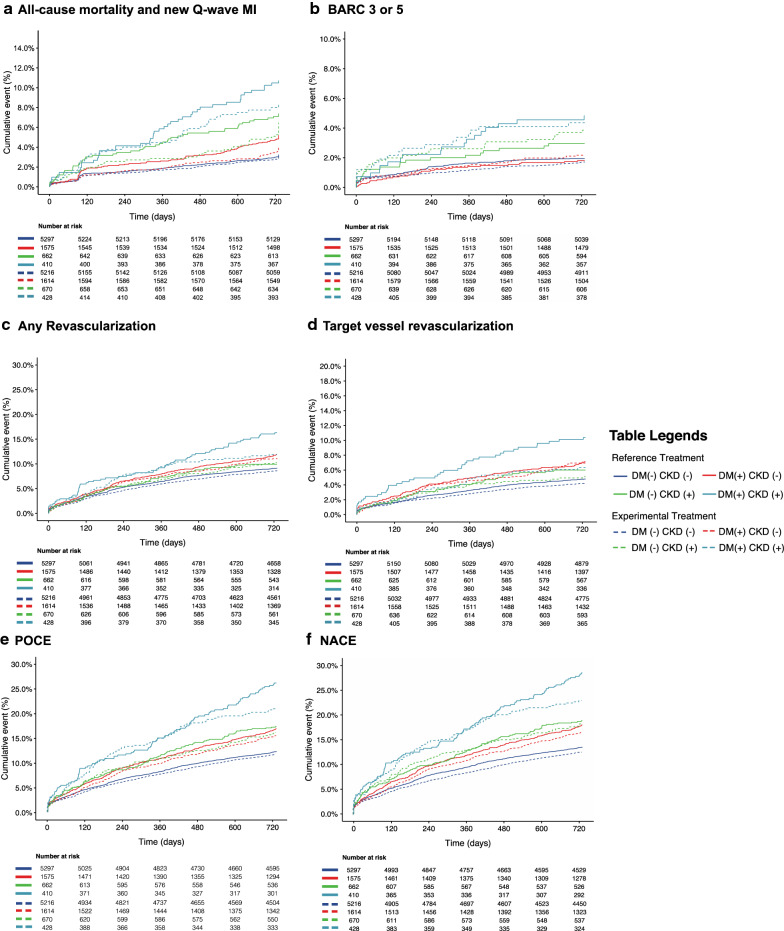
Kaplan–Meier curves showing the clinical events according to treatment regimen and DM/CKD status. **a** All-cause mortality and new Q-wave MI; **b** Bleeding Academic Research Consortium (BARC)–defined type 3 or 5 bleeding events; **c** Any revascularization; **d** Target vessel revascularization; **e** POCE; **f** NACE;

Compared with the reference regimen (DAPT for 12 months and then aspirin for 12 months), the experimental regimen (DAPT for 1 month followed by ticagrelor monotherapy for 23 months) did not show lower rates of the primary or the key safety secondary endpoints in DM + /CKD + patients, or in any of the other three subgroups (Fig. 3a, b, and Table 3). Although not statistically significant, the absolute risk reduction of the primary endpoint gradually increased in the following order of DM-/CKD-, DM-/CKD + , DM + /CKD-, DM + /CKD + (0.3%, 1.0%, 1.1%, and 2.3%) in patients receiving the experimental regimen.

**Table 3:**
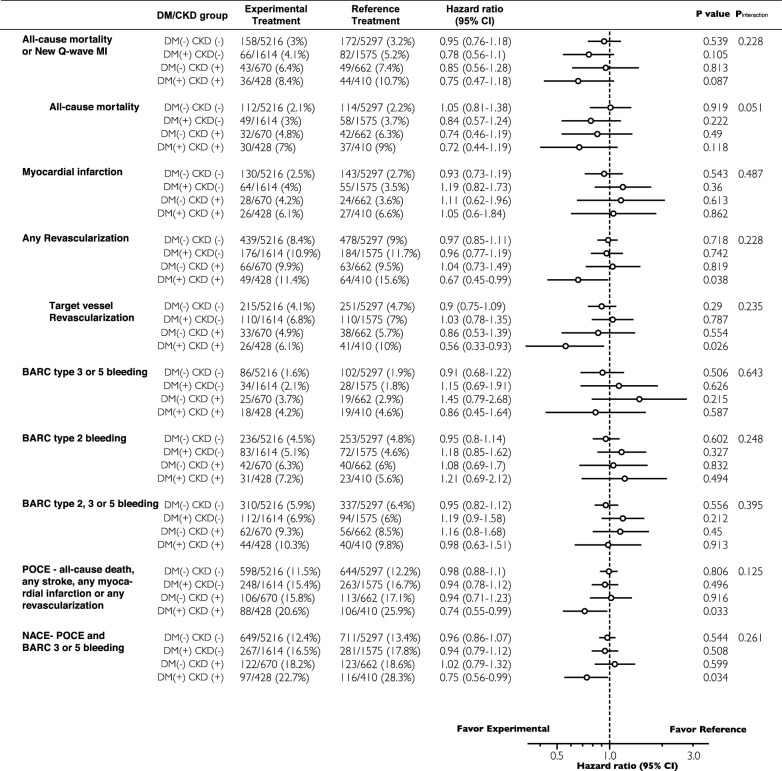
Forest plot of the endpoints according to treatment regimen and DM/CKD status

Adjusted to age, sex, body mass index (BMI), clinical presentation (ACS versus stable CAD), stroke, peripheral vascular disease (PVD), chronic obstructive pulmonary disease (COPD), previous PCI, hypercholesterolemia, hypertension, current smoking status, complex PCI, ACEI or ARB, beta-blockade, statin, Paris thrombotic risk score, and Paris bleeding risk score

Among the DM + /CKD + patients, the experimental regimen was associated with lower rates of POCE (20.6% versus 25.9%, HR 0.74; 95%CI [0.55–0.99], p = 0.043, p_interaction_ = 0.155) and NACE (22.7% versus 28.3%, HR 0.75; 95%CI [0.56–0.99], p = 0.044, p_interaction_ = 0.310), which were mainly driven by lower rates of any revascularization (11.5% versus 15.6%; adjusted HR 0.67; 95%CI [0.45–0.99], P = 0.042, p_interaction_ = 0.286) and TVR (6.1% versus 10.0%; adjusted HR 0.56; 95%CI [0.33–0.93], P = 0.026, p_interaction_ = 0.238; Fig. 3c–f, and Table 3), as compared with the reference regimen. The numbers needed-to-treat to reduce a POCE, NACE, any revascularization and TVR event were 19, 18, 24 and 25, respectively. Additional sensitivity analyses were performed to investigated the treatment effects of experimental regimen in patients who were adherent to the allocated medications, in ACS patients, and in Stable CAD patients, respectively. The results are shown in Additional file 1: Table S5–S7.

### Landmark analysis

Given that according to the study protocol, the reference treatment strategy arm received conventional 12-month DAPT (Clopidogrel/Ticagrelor was stopped at 12-month) followed by 12- month aspirin monotherapy, we performed a landmark analysis at 365 days after the index procedure to specifically analyze the impact of P2Y12 discontinuation in the reference strategy. The results showed that among DM + /CKD + patients, between 0–365 days after randomization, the experimental and reference regimen had similar rates of all investigated endpoints (Additional file 1: Table S4 and Fig. S2 ), whereas between 365–730 days after randomization, compared with the reference regimen, the experimental regimen was associated with significantly lower rates of POCE (5.8% versus 11.0%, HR 0.49; 95% CI [0.29–0.82], p = 0.007, p_interaction_ = 0.040), NACE (5.8% versus 11.2%, HR 0.48; 95% CI [0.29–0.82], p = 0.007, p_interaction_ = 0.013), any revascularization (2.3% versus 6.6%, adjusted HR 0.29; 95% CI [0.13–0.65], P = 0.003, p_interaction_ = 0.056) and TVR (1.4% versus 2.9%, adjusted HR 0.29; 95% CI [0.09–0.91], P = 0.033, p_interaction_ = 0.112) (Table 4 and Additional file 1: Fig. S2). The rate of BARC type 3 or 5 bleeding events (0.7% versus 1.5%, P = 0.331) was similar between the two antiplatelet regimens between 365 and 730 days after randomization.

**Table 4:**
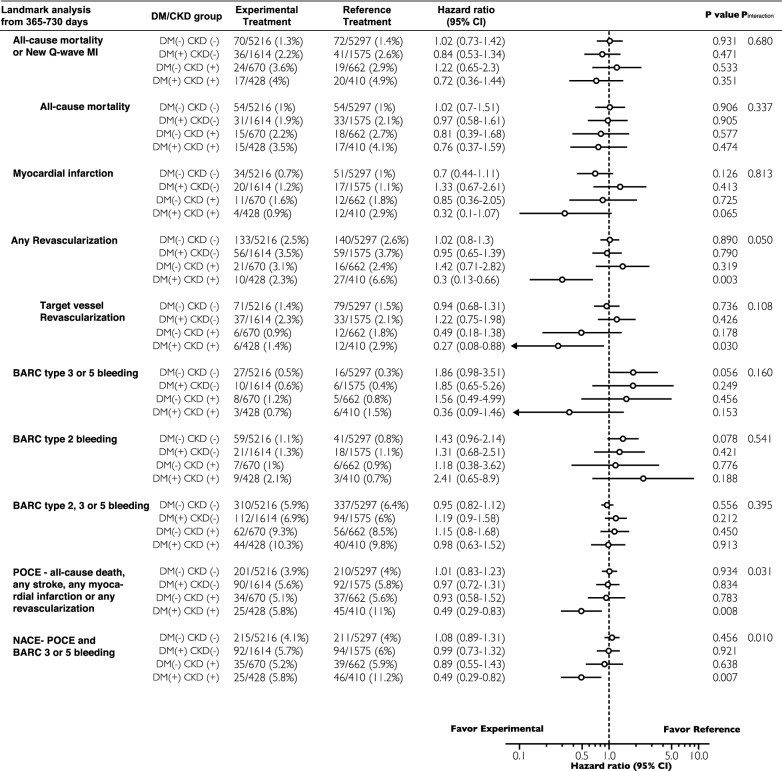
Forest plot of the endpoints by landmark analyses (365-730 days)

## Discussion

The main findings of this post hoc analysis of the GLOBAL LEADERS trial can be summarized as follows:

1. The concomitant presence of DM and CKD is not uncommon in an “all-comers” trial, representing 21% of the patients with DM, and 5% of the overall study population.
2. Up to two years post-PCI, there was a gradient in the thrombotic and bleeding risk among patients stratified according to the presence or absence of DM or CKD, with the highest risk found among subjects having both comorbidities.
3. In patients with both DM and CKD, the primary endpoint (all-cause mortality or new Q-wave MI) or the key safety secondary endpoint (BARC type 3 or 5 bleeding) did not differ significantly between the experimental and the reference regimens. Notwithstanding, the experimental regimen was associated with lower rates of POCE and NACE, mainly driven by repeat revascularization.

### The prevalence and prognosis of CAD patients with DM and CKD

Both DM and CKD are independently associated with an increased risk of cardiovascular ischemic events, which can be attributed to patients’ pro-thrombotic and pro-inflammatory status [2, 3]. These two risk factors of coronary heart disease have also been shown to synergistically amplify the hazards when they co-exist. Reports published nearly two decades ago showed that mortality rates one year after successful PCI in DM patients with moderate and severe CKD were respectively, 5- and 12-times higher when compared to patients with normal renal function [21]. A subgroup analysis of the PLATO trial -a trial conducted over a decade ago [22], showed that patients with the combination of DM and CKD had a greater than threefold increase in the risk of mortality [6]. In the contemporary GLOBAL LEADERS trial, we found that despite the progressive improvements in stent design and secondary preventive pharmacotherapies, patients with both DM and CKD still had a 2.1-fold higher risk of mortality, 1.6-fold higher risk of repeat revascularization, and 1.6-fold higher risk of BARC 3 or 5 bleeding, compared with patients without these risk factors. Although these results suggest that the hazards of having both comorbidities have somewhat attenuated over the years, patients with both DM and CKD were still at high risk of ischemic and bleeding events. These observations underscore the need to identify novel therapeutic approaches that can reduce the risks in this specific population.

In the current analysis, we found that 20.8% (838/4027) of DM patients had CKD. This proportion is relatively similar among some pivotal cardiovascular RCTs. For instance, in the PLATO trial that investigated adjunctive antiplatelet pharmacotherapy in patients with acute coronary syndromes [6], the percentage of DM patients who had CKD was 22.0% (1058/3807). In the SYNTAX trial, which tested the optimal revascularization technique in patients with complex coronary lesions, the proportion was 20.8% (85/408, unpublished data). However, data from a German national database [23] and two dedicated registries (*Diabetes*-*Patienten*-*Verlaufsdokumentation* and *DIabetes Versorgungs-Evaluation*) [24] suggested that approximately 40–50% of individuals with DM have comorbid CKD. Therefore, when compared with these population-based studies, the DM + /CKD + population in the GLOBAL LEADERS and some cardiovascular RCTs might be underrepresented, or conversely these registries with specific inclusion criteria may have an overrepresentation of the syndrome.

### DAPT strategy for DM + /CKD + patients (0–1 year post PCI)

The optimal DAPT strategy for DM + /CKD + patients remains a matter of debate owing to scarce evidence. Generally, DM + /CKD + patients are at high bleeding risk [25]. In the GLOBAL LEADERS population, 71.0% of the DM + /CKD + patients had a PRECISE-DAPT score of 25 or more. As suggested by the 2018 European Society of Cardiology guidelines on Myocardial Revascularization [1], patients with high bleeding risk (PRECISE-DAPT score of 25 as the cutoff point) should discontinue DAPT after 3- (in stable CAD) or 6-months (in ACS) post-PCI to reduce the risk of bleeding; however, DM + /CKD + patients were also at high thrombotic risk (73.4% of these patients had a Paris thrombotic risk score of > 5). Indeed, a short DAPT strategy would reduce bleeding events, but at the same time, might plausibly augment the thrombotic risk [26, 27].

Considering the dilemma of DAPT duration, the strategy of ticagrelor monotherapy has been proposed as a means to reduce the risk of bleeding while maintaining a similar risk of thrombotic events after PCI. The TWILIGHT trial [8, 28], in which either DM or CKD constituted an enrichment criteria according to the protocol (2620 pts with DM and 1145 pts with CKD in the TWILIGHT trial), has compared 3-month DAPT followed by 12-month ticagrelor monotherapy after PCI with standard DAPT strategy. The results showed a significant reduction of BARC type 2, 3 or 5 bleeding events in the ticagrelor monotherapy arm, while demonstrating a similar risk of the composite secondary endpoint of all-cause death, non-fatal MI, or stroke. Compared with the TWILIGHT trial, the current study showed that in DM + /CKD + patients, ticagrelor monotherapy strategy in the first year had similar rates of all-cause mortality, MI or revascularization, as well as the rate of BARC type 2, 3 or 5 bleeding events, compared with the standard DAPT strategy. These results showed that although the thrombotic risks were higher in DM + /CKD + patients, ticagrelor monotherapy (or the “aspirin-free strategy”) might not be associated with increased thrombotic events compared with the standard DAPT.

### Prolonged ticagrelor monotherapy for secondary prevention (1 year post PCI)

To date, there is no evidence elaborating the optimal antiplatelet medication for the secondary prevention of the DM + /CKD + patients post PCI. Alike other patients, those patients are now generally treated with aspirin lifelong for secondary prevention. Whether ticagrelor represents a worse, alternative, or better choice still debatable. In DM patients with stable CAD and a history of PCI), results of the THEMIS-PCI trial [29, 30] have demonstrated that compared to aspirin for secondary prevention, ticagrelor reduced the ischemic endpoint of cardiovascular death, MI, and stroke with modestly increasing the bleeding events. In total, ticagrelor improved the net clinical benefit (9.3% versus 11.0%, HR = 0.85, 95% CI 0.75–0.95, p = 0.005) in the THEMIS-PCI population. For the DM + /CKD + patients, whether it is legitimate to simply apply to the recommendation for DM patients (such as the results of the TWILIGHT and the THEMIS-PCI trial), is somehow based on empirical experiences. So far, there is no specific narrative in the consensus or guideline helping the clinician to make the decision. The current analysis found that in DM + /CKD + patients, compared with aspirin monotherapy, the ticagrelor monotherapy had similar BARC type 2, 3 or 5 bleeding events, meanwhile, was associated with lower rates of POCE and NACE, which were predominantly confined to reductions in any revascularization or TVR events that occurred during the second year of the trial.

Legitimately, like the THEMIS-PCI trial, prolonging the use of ticagrelor would increase the risk of bleeding. The neutral statistical findings in our analysis regarding BARC type 2, 3 or 5 bleeding events might be due to play of chance or the relatively low sample size (although it is one of the largest trials investigating such issue). However, the improved net clinical benefit of the ticagrelor monotherapy shown in our analysis and the THEMIS-PCI trial supported that prolonged ticagrelor might be a reasonable treatment option for DM or DM + /CKD + patients regarding secondary prevention. Of note, given the inherent limitations of sub-analyses, our findings cannot make strong inferences nor necessitate changes in clinical practice.

## Limitations

The following limitations have to be considered in the present analysis. (1) Given that the two antiplatelet strategies did not differ significantly with regard to rates of the primary endpoint in the overall trial [7], and the post hoc nature of the study, all reported analyses have to be considered strictly exploratory. (2) The randomization in the GLOBAL LEADERS trial was not stratified according to the presence of DM or CKD, therefore some imbalances between the randomized groups may exist among the four sub-categories. Although multivariable adjusted Cox proportional hazard models were performed to try to estimate the true treatment effects of the different regimens, the usual deficiency for observational studies exists, such as the inability to include all relevant confounders especially those unmeasured, causing bias which cannot be adjusted.

## Conclusions

The present analysis showed that in a contemporary PCI cohort, patients with DM and CKD are at markedly increased risk of long-term thrombotic and bleeding events, compared with patients one or neither of these risk factors. In patients with both comorbidities, ticagrelor monotherapy was not associated with a lower rate of the primary endpoint (all-cause mortality or new Q-wave MI) or bleeding (BARC type 3 or 5 bleeding), but was associated with a lower rate of POCE and NACE, which was mainly driven by the lower rate of any revascularization.

## Supplementary information

**Additional file 1: Table S1.** Forest plot of the ischemic endpoints according to treatment regimen and DM/CKD status. **Table S2.** Forest plot of the bleeding endpoints according to treatment regimen and DM/CKD status. **Table S3.** Forest plot of sensitivity analyses (stage II to V CKD by KDIGO classification) showing outcomes according to treatment regimen and DM/CKD status. **Table S4.** Forest plot of landmark analyses (0–365 days) showing outcomes of reference versus experimental treatment according to DM/CKD status. **Table S5.** Forest plot of sensitivity analyses (subjects who were adherent to the allocated medication) showing outcomes according to treatment regimen and DM/CKD status. **Table S6.** Forest plot of sensitivity analyses (ACS patients) showing outcomes according to treatment regimen and DM/CKD status. **Table S7.** Forest plot of sensitivity analyses (Stable CAD patients) showing outcomes according to treatment regimen and DM/CKD status. **Table S8.** Forest plot of sensitivity analyses (Propensity score adjusted Cox regression model) showing outcomes according to treatment regimen and DM/CKD status. **Figure S1.** Distribution of propensity score. **Figure S2.** Kaplan-Meier curves of the landmark analysis showing outcomes of treatment regimen according to DM/CKD status.

## Data Availability

The datasets generated and/or analysed during the current study are not publicly available but are available from the corresponding author on reasonable request.

## Abbreviations

ACS: Acute coronary syndrome
ARC: Academic Research Consortium
BARC: Bleeding Academic Research Consortium
BMI: Body mass index
CABG: Coronary artery bypass graft surgery
CAD: Coronary artery disease
CKD: Chronic Kidney Disease
COPD: Chronic Obstructive Pulmonary Disease
DAPT: Dual-antiplatelet therapy
DES: Drug Eluting Stent
DM: Diabetes Mellitus
ECG: Electrocardiogram/electrocardiography
eGFR: Estimated glomerular filtration rate
MDRD: Modification of Diet in Renal Disease
NACE: Net adverse clinical events
NSTEMI: Non-ST-segment elevation MI
PCI: Percutaneous Coronary Intervention
POCE: Patient-oriented Composite Endpoint
ST: Stent thrombosis
STEMI: ST elevation MI
TIMI: Thrombolysis In Myocardial Infarction
TLF: Target Lesion Failure
TV MI: Target vessel Myocardial Infarction
TVR: Target vessel revascularization
