# Supplementary material for: Ticagrelor monotherapy in patients with concomitant diabetes mellitus and chronic kidney disease: a post hoc analysis of the GLOBAL LEADERS trial
Source: Cardiovasc Diabetol. 2020 Oct 16;19:179. doi: 10.1186/s12933-020-01153-x (PMC7568378; doi:10.1186/s12933-020-01153-x)
Supplement: Supplementary file 1 — Additional file 1: Table S1. Forest plot of the ischemic endpoints according to treatment regimen and DM/CKD status. Table S2. Forest plot of the bleeding endpoints according to treatment regimen and DM/CKD status. Table S3. Forest plot of sensitivity analyses (stage II to V CKD by KDIGO classification) showing outcomes according to treatment regimen and DM/CKD status. Table S4. Forest plot of landmark analyses (0–365 days) showing outcomes of reference versus experimental treatment according to DM/CKD status. Table S5. Forest plot of sensitivity analyses (subjects who were adherent to the allocated medication) showing outcomes according to treatment regimen and DM/CKD status. Table S6. Forest plot of sensitivity analyses (ACS patients) showing outcomes according to treatment regimen and DM/CKD status. Table S7. Forest plot of sensitivity analyses (Stable CAD patients) showing outcomes according to treatment regimen and DM/CKD status. Table S8. Forest plot of sensitivity analyses (Propensity score adjusted Cox regression model) showing outcomes according to treatment regimen and DM/CKD status. Figure S1. Distribution of propensity score. Figure S2. Kaplan-Meier curves of the landmark analysis showing outcomes of treatment regimen according to DM/CKD status. [file 12933_2020_1153_MOESM1_ESM.docx]

**SUPPLEMENTAL MATERIAL**

**This appendix has been provided by the authors to give readers additional information about their work.**

**Contents**

[Supplement Methods 2](#_Toc51168943)

[Supplement Table 1 Forest plot of the ischemic endpoints according to treatment regimen and DM/CKD status 3](#_Toc51168944)

[Supplement Table 2 Forest plot of the bleeding endpoints according to treatment regimen and DM/CKD status 4](#_Toc51168945)

[Supplement Table 3 Forest plot of sensitivity analyses (stage II to V CKD by KDIGO classification) showing outcomes according to treatment regimen and DM/CKD status 5](#_Toc51168946)

[Supplement Table 4 Forest plot of landmark analyses (0-365 days) showing outcomes of reference versus experimental treatment according to DM/CKD status 6](#_Toc51168947)

[Supplement Table 5 Forest plot of sensitivity analyses (subjects who were adherent to the allocated medication) showing outcomes according to treatment regimen and DM/CKD status 7](#_Toc51168948)

[Supplement Table 6 Forest plot of sensitivity analyses (ACS patients) showing outcomes according to treatment regimen and DM/CKD status 8](#_Toc51168949)

[Supplement Table 7 Forest plot of sensitivity analyses (Stable CAD patients) showing outcomes according to treatment regimen and DM/CKD status 9](#_Toc51168950)

[Supplement Table 8 Forest plot of sensitivity analyses (Propensity score adjusted Cox regression model) showing outcomes according to treatment regimen and DM/CKD status 10](#_Toc51168951)

[Supplement Figure 1 Distribution of propensity score 11](#_Toc51168952)

[Supplement Figure 2 Kaplan-Meier curves of the landmark analysis showing outcomes of treatment regimen according to DM/CKD status 12](#_Toc51168953)

[Reference 13](#_Toc51168954)

# Supplement Methods

Propensity scores (PS) were calculated [1] by including the variables of demographic characteristics (age, sex, BMI), ACS/stable CAD, coexisting medical conditions (diabetes, insulin-dependent diabetes, hypertension, hypercholesterolemia, current smoker, previous bleeding, stroke, peripheral vascular disease, chronic obstructive pulmonary disease, renal failure, myocardial infarction, PCI, or CABG), antiplatelet therapy, Paris bleeding risk score, Paris thrombotic risk score, and medication on discharge (ACEI or ARB, beta-blockade, and statin) and complex PCI.

PS were distributed in a range of 0.405 to 0.553. Therefore, no case was considered to have an extreme propensity score, and none were trimmed. The distribution of PS is shown in Supplement Figure 1.

In order to calculate PS, all missing data were filled in the database by multiple imputations [2]. There was no variable with data missing more than 5%, therefore all missing data were considered as missing completely at random (MCAR).

# Additional Table S1 Forest plot of the ischemic endpoints according to treatment regimen and DM/CKD status

Adjusted to age, sex, body mass index (BMI), clinical presentation (ACS versus stable CAD), stroke, peripheral vascular disease (PVD), chronic obstructive pulmonary disease (COPD), previous PCI, hypercholesterolemia, hypertension, current smoking status, complex PCI, ACEI or ARB, beta-blockade, statin, Paris thrombotic risk score, and Paris bleeding risk score

# Table S2 Forest plot of the bleeding endpoints according to treatment regimen and DM/CKD status

# Table S3 Forest plot of sensitivity analyses (stage II to V CKD by KDIGO classification) showing outcomes according to treatment regimen and DM/CKD status

# Table S4 Forest plot of landmark analyses (0-365 days) showing outcomes of reference versus experimental treatment according to DM/CKD status

# Table S5 Forest plot of sensitivity analyses (subjects who were adherent to the allocated medication) showing outcomes according to treatment regimen and DM/CKD status

# Table S6 Forest plot of sensitivity analyses (ACS patients) showing outcomes according to treatment regimen and DM/CKD status

# Table S7 Forest plot of sensitivity analyses (Stable CAD patients) showing outcomes according to treatment regimen and DM/CKD status

# Table s8 Forest plot of sensitivity analyses (Propensity score adjusted Cox regression model) showing outcomes according to treatment regimen and DM/CKD status

# Figure S1 Distribution of propensity score


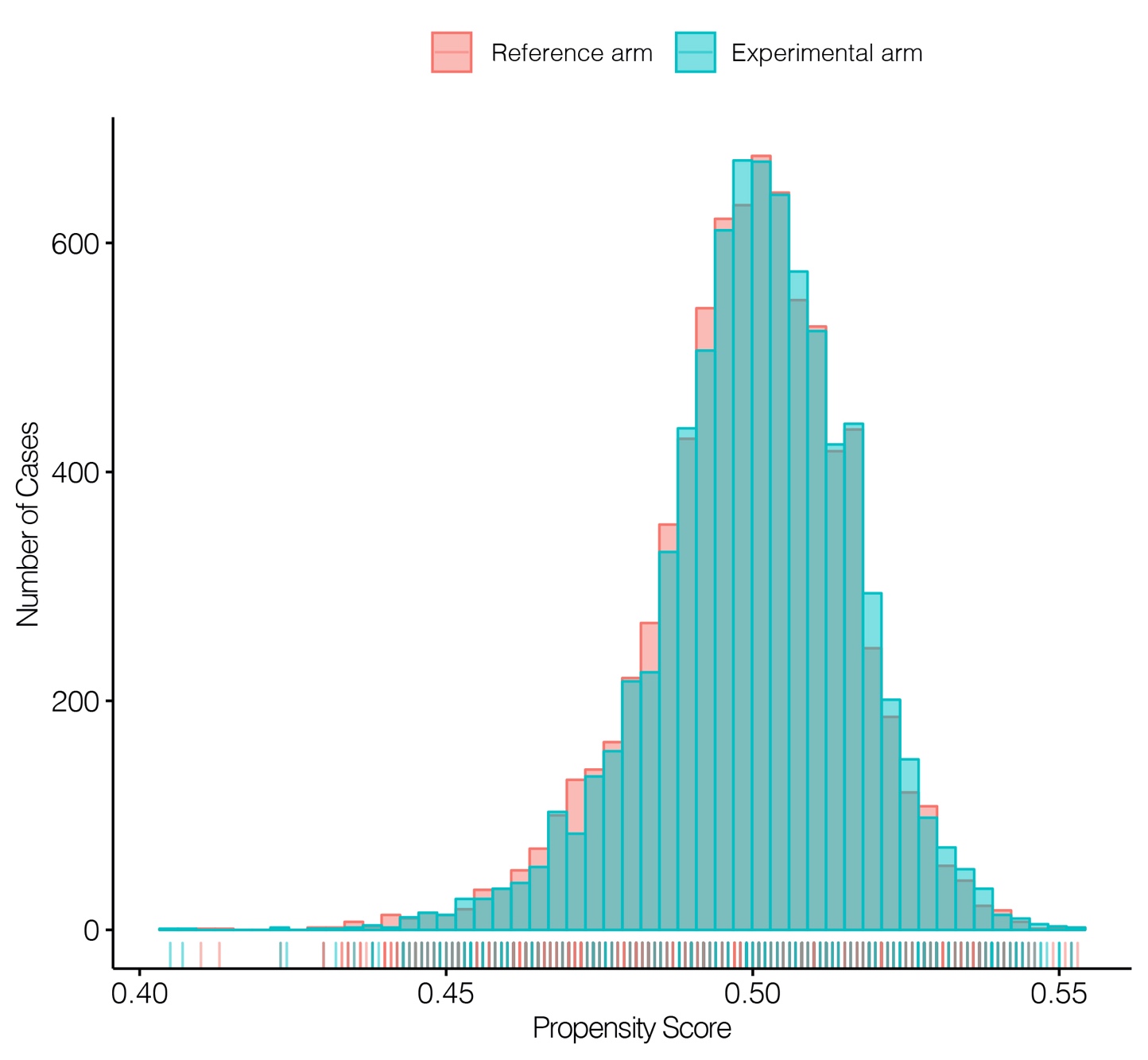


# Figure S2 Kaplan-Meier curves of the landmark analysis showing outcomes of treatment regimen according to DM/CKD status

# Reference

1. Elze MC, Gregson J, Baber U, Williamson E, Sartori S, Mehran R, Nichols M, Stone GW, Pocock SJ: **Comparison of Propensity Score Methods and Covariate Adjustment: Evaluation in 4 Cardiovascular Studies**. *J Am Coll Cardiol* 2017, **69**(3):345-357.

2. Little RJA, Rubin DB: **Statistical analysis with missing data**: John Wiley & Sons, Inc.; 1986.
